# Advancing DBD Plasma Chemistry: Insights into Reactive Nitrogen Species such as NO_2_, N_2_O_5_, and N_2_O Optimization and Species Reactivity through Experiments and MD Simulations

**DOI:** 10.1021/acs.est.4c04894

**Published:** 2024-08-29

**Authors:** Masoom Shaban, Nina Merkert, Adri C. T. van Duin, Diana van Duin, Alfred P. Weber

**Affiliations:** † Institute of Particle Technology, Clausthal University of Technology, 38640 Clausthal-Zellerfeld, Germany; ‡ Institute of Applied Mechanics, Clausthal University of Technology, 38640 Clausthal-Zellerfeld, Germany; § Department of Mechanical Engineering, Pennsylvania State University, University Park, Pennsylvania 16802, United States; ∥ RxFF Consulting LLC, 1524 West College Avenue, Suite 202, State College, Pennsylvania 16801, United States

**Keywords:** DBD plasma, aerosol polymerization, molecular dynamics (MD) simulations, ReaxFF, reactive nitrogen species (RNS), OH, H_2_O_2_, NO_2_, N_2_O_5_, N_2_O

## Abstract

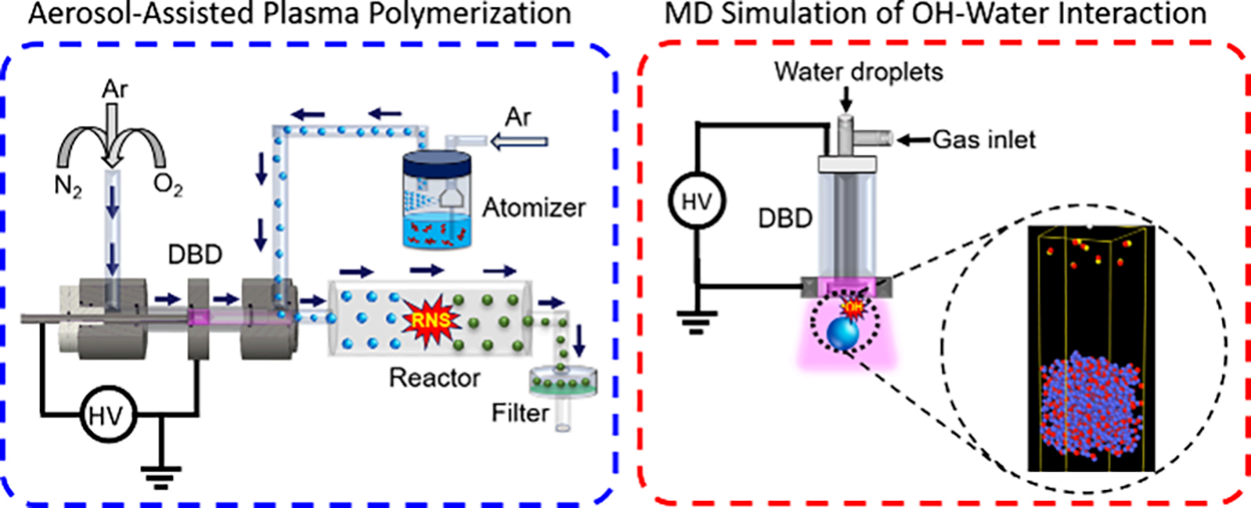

This study aims to fine-tune the plasma composition with a particular emphasis on reactive nitrogen species (RNS) including nitrogen dioxide (NO_2_), dinitrogen pentoxide (N_2_O_5_), and nitrous oxide (N_2_O), produced by a self-constructed cylindrical dielectric barrier discharge (CDBD). We demonstrated the effective manipulation of the plasma chemical profile by optimizing electrical properties, including the applied voltage and frequency, and by adjusting the nitrogen and oxygen ratios in the gas mixture. Additionally, quantification of these active species was achieved using Fourier transform infrared spectroscopy. The study further extends to exploring the aerosol polymerization of acrylamide (AM) into polyacrylamide (PAM), serving as a model reaction to evaluate the reactivity of different plasma-generated species, highlighting the significant role of NO_2_ in achieving high polymerization yields. Complementing our experimental data, molecular dynamics (MD) simulations, based on the ReaxFF reactive force field potential, explored the interactions between reactive oxygen species, specifically hydroxyl radicals (OH) and hydrogen peroxide (H_2_O_2_), with water molecules. Understanding these interactions, combined with the optimization of plasma chemistry, is crucial for enhancing the effectiveness of DBD plasma in environmental applications like air purification and water treatment.

## Abstract 2

This research advances plasma technology for efficient nitrogen oxide production, with significant implications for environmental remediation, including air quality improvement and water decontamination.

## Introduction

Cold atmospheric plasma (CAP) is a unique form of ionized gas where high-temperature electrons are in non-equilibrium with low-temperature ions and molecules. This technology employs nonthermal plasma to generate a range of reactive species under ambient conditions, making it suitable for applications that require precise and controlled chemical reactions. Over the past two decades, research on CAP for indoor air purification, removing pollutants from exhaust gases, food processing, and water treatment has been prevalent.^1−10^

There has been significant interest in reactive nitrogen species (RNS) such as NO_2_, N_2_O_5_, and N_2_O for their broad applications in environmental settings. For example, N_2_O_5_, with its high solubility, is suitable for water treatment by neutralizing contaminants.^11^ Similarly, NO_2_ plays a vital role in antimicrobial applications, inactivating a wide range of pathogens and providing a nontoxic method for sterilization and disinfection,^12^ which is especially important for maintaining hygiene in food and water safety without compromising quality.

Although most research reports successful applications of cold plasma generated by dielectric barrier discharge (DBD), only a limited number of studies have explored the fundamental aspects, such as the effects of gas composition and electrical adjustments on plasma discharge.^13−17^ For example, Höft et al.^18^ investigated the effect of oxygen content on the spatial and temporal behavior of pulse-driven DBD in N_2_/O_2_ gas mixtures. They found that increasing the O_2_ content impacted the discharge characteristics, resulting in a shorter discharge duration, an expanded discharge radius, and a higher plasma current. Similarly, Guerra et al.^19^ investigated the impact of the N_2_(A^3^Σ_u_^+^) metastable state on stationary N_2_ and N_2_–O_2_ discharges using a kinetic model. They found that the presence of small amounts of O_2_ in a nitrogen discharge quenches the N_2_ metastable state through interactions with O, O_2_, and NO, which significantly impacts the NO formation and discharge characteristics. Schmidt-Bleker et al.^17^ studied the reactive species generated by the commercial plasma jet kinpen Sci using Fourier transform infrared spectroscopy (FTIR) and kinetic simulations. They found that varying the shielding gas composition and humidity levels affected the densities of ozone and nitrogen dioxide. Moreover, Kogelschatz advanced dielectric-barrier discharges (DBDs) for industrial use, improving efficiency and scalability in applications like ozone generation and plasma displays through innovative power electronics and discharge physics.^20,21^ Expanding on these foundational studies, further investigations are needed to examine the fundamental behavior of chemical products with different Ar/N_2_/O_2_ gas mixture ratios and the electrical properties of the DBD, including applied voltage, frequency, and discharge power. Therefore, this work focuses on identifying and characterizing plasma chemistry to optimize conditions for producing reactive nitrogen species (RNS). By providing a detailed method for regulating RNS production, this research aims to enhance the efficiency and effectiveness of plasma technology in various environmental and industrial applications.

Additionally, to apply plasma technology for inactivating aerosolized pathogens and purifying the air, it is crucial to engineer methods that can effectively neutralize microorganisms in water-based environments.^22^ This is important because most microorganisms thrive in moist conditions and are often protected by a liquid film.^23^ Understanding the interaction between plasma species and the surrounding liquid layers is critical, because reactive plasma species might penetrate the liquid layer to directly interact with biomolecules, or they might undergo transformations within the liquid layer, leading to the formation of new species.^22^ Furthermore, studying the interaction between plasma-generated species and water molecules can enhance our understanding of the mechanisms behind plasma-activated water, thereby improving its applications in water treatment, air pollution control, and soil remediation.^24,25^ Molecular dynamics (MD) simulations are well suited to investigate these interactions at an atomic level. They simulate the microenvironment surrounding the microorganisms, including the moisture layers, and introduce plasma species into this setting. This approach allows for a detailed examination of the interactions between the plasma species and liquid layers. However, relatively few modeling studies have been conducted so far to examine the atomic-level interactions of reactive plasma species with water and biomolecules.^22,26^ Therefore, in the second part of this work, we used atomistic simulations to examine the interactions between plasma species and water. We concentrated on the OH radical and its reaction products with water, as the short lifespan of the OH radical presents significant challenges for experimental investigation and characterization. To apply the plasma-generated OH radicals for experimental applications, it is crucial to generate this species near its intended target, which complicates their use in practical applications. To address this limitation, we employed MD simulations to investigate the behavior of OH radicals in water, offering insights into their reactivity and interaction mechanisms at the atomic level. These simulations enhance our understanding of how to stabilize OH radicals in water, thereby extending their effective lifespan for practical applications.

## Experimental Setup

**Figure 1:**
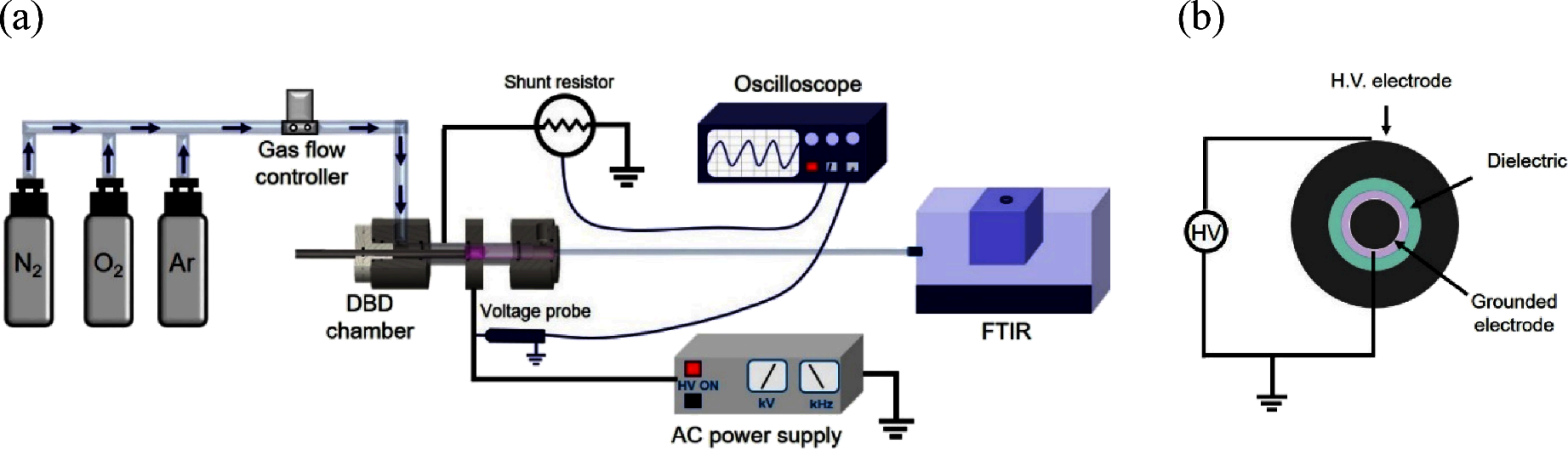
(a) Schematic representation of the experimental setup and (b) cross-sectional view of the CDBD utilized in our study.

Figure 1 illustrates the experimental setup designed for the generation and analysis of reactive plasma species using a CDBD system.^27^ The design displays a cylindrical assembly that centers on two key components: electrodes made of stainless steel, with the inner electrode taking the shape of a disc and the outer electrode forming a surrounding ring. The inner electrode, grounded for safety and stability, has a diameter of 12 mm. The outer electrode, connected to a high voltage to initiate the discharge, is constructed as a ring with a significant thickness of 16 mm, providing robust structural support and enabling a uniform electric field distribution essential for the CDBD process. The electrodes are spatially arranged with the aid of a glass dielectric tube, which has an inner diameter of 13 mm and a wall thickness of 1.5 mm. This dielectric not only insulates the electrodes but also serves as the structural gas channel. Plasma is generated within the narrow 0.5 mm ring-shaped gap that exists between the metal disc and the inner wall of the dielectric tube. This configuration is fundamental to CDBD operation, as it facilitates the generation of a consistent and stable plasma, which is critical for the production of the reactive species and subsequent analysis. The CDBD was operated with 70 vol % Ar and 30 vol % admixture of N_2_ and O_2_ at varying mixture ratios (gas 5.0; gas purity: 99.999%). The oxygen vol % was adjusted from 0 to 30%, thereby spanning the range from pure N_2_ to pure O_2_.

As illustrated in Figure 1, a power generator (Plasma Generator G2000, Redline technology), designed to deliver adjustable sinusoidal voltage, was used for applying a high voltage to the outer electrode. The electrical parameters under these settings were analyzed using an oscilloscope from Rhode & Schwarz. The study focused on observing changes in voltage and current over time, along with how the discharge power varied with different applied voltages to understand the electrical behavior of the CDBD reactor better. The applied voltage, critical for initiating the discharge, was meticulously controlled via the power supply and measured with a high voltage probe from Tektronix, model P6015A 1000x, due to the requirement for kilovolt-level voltages. A shunt resistor of 50 ohms was installed in series with the CDBD circuit between the high voltage and grounded electrodes (Figure 1). The primary aim of this configuration was to enable the calculation of the current flowing through the CDBD by measuring the voltage drop across the shunt with an oscilloscope and applying Ohm’s law.

Qualitative analysis of the species produced by the plasma was conducted by using a Fourier transform infrared spectrometer (Bruker Tensor 27). This instrument is designed to detect absorption in the mid-infrared range, spanning from 4000 to 400 cm^–1^, and is integrated with a gas absorption cell for sample analysis. The infrared absorption spectra obtained were analyzed to determine and quantify the reactive species. This was achieved by comparing the measured spectra with reference spectra from the HITRAN database and the PNNL Quantitative Infrared Database, as outlined in previous studies.^28−33^

## Experimental Results and Discussion

### Discharge Power Calculation

In our study, the power characteristics of the CDBD were calculated by analyzing the measured voltage and current waveforms. The instantaneous power *P*(*t*) was calculated as the product of the instantaneous voltage *V*(*t*) and current *I*(*t*) using the formula:
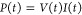
For a more comprehensive assessment of the energy consumption of the CDBD during prolonged operations, the average power *P̅* was calculated by integrating the instantaneous power across several cycles, spanning *n* periods in total. This method provides an in-depth analysis of the efficiency of CDBD over an extended duration.
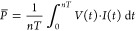


**Figure 2:**
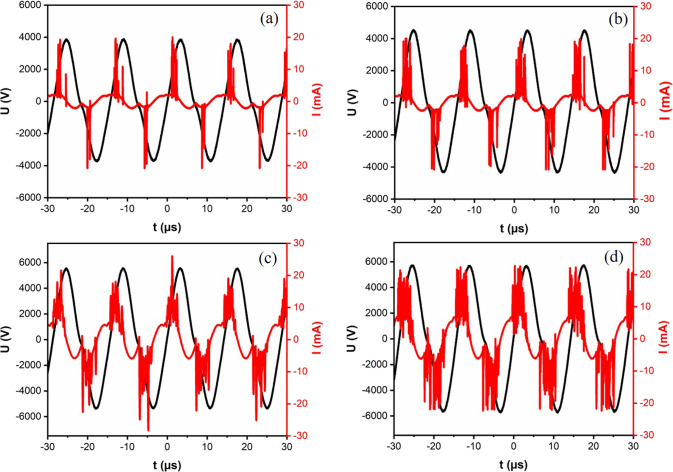
Voltage and current waveforms of the CDBD reactor operating at a constant frequency of 40 kHz, depicted at various settings: (a) Upp = 7.9, *P* = 1 W, (b) Upp = 9, *P* = 3 W, (c) Upp = 10.6, *P* = 5 W, and (d) Upp = 11.6, *P* = 7 W.

To determine the optimal conditions for NO_*x*_ production, different discharge powers were employed. For instance, Figure 2 displays four distinct voltage and current waveforms at a constant frequency of 40 kHz. The voltage waveform, depicted in black, demonstrates sinusoidal behavior, while the current waveform, illustrated in red, manifests oscillations that exhibit a predictable phase shift in relation to the voltage. The current waveform features pulses that reflect microdischarge events within the plasma. The result shows that the power consumption of the CDBD increases with the applied voltage; as the voltage rises, the electric field strength across the dielectric barrier increases, leading to more energetic and numerous microdischarges. Consequently, this elevates ionization and excitation processes within the CDBD and increases power consumption.

### Regulating Plasma Chemistry through the Adjustment of CDBD Power

**Figure 3:**
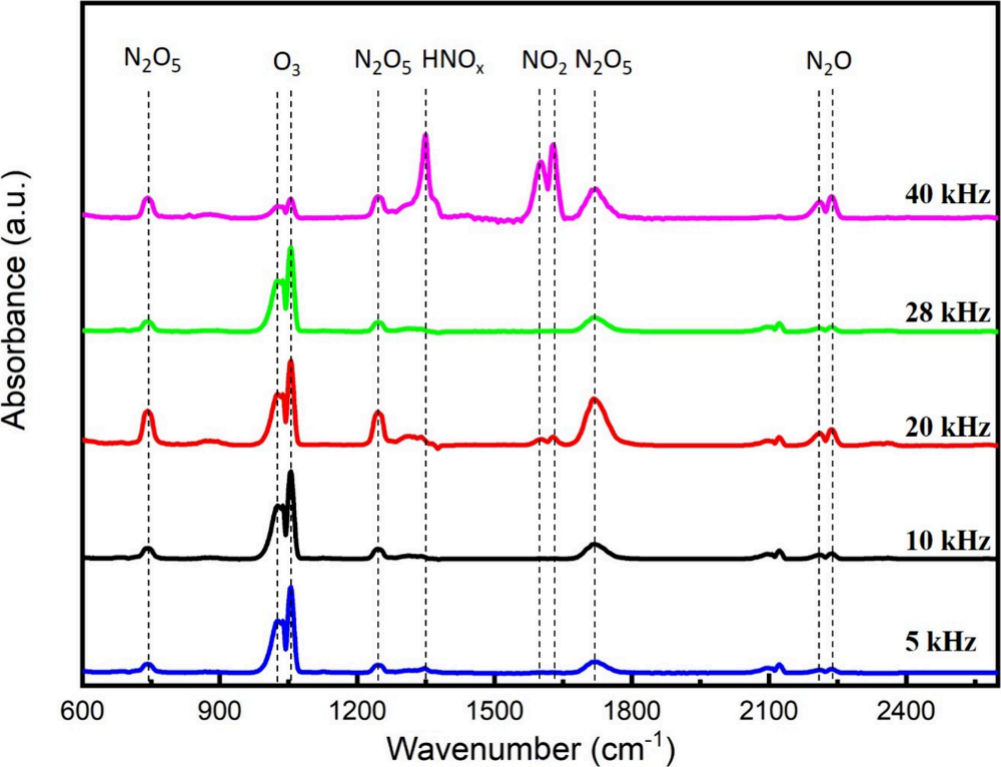
Gas-phase FTIR spectra of CDBD in the gas mixture consisting of 70 vol % Ar, 25 vol % N_2_, and 5 vol % O_2_ (total flow rate: 80 L/h) at various applied frequencies.

To determine the ideal electrical parameters for the CDBD that maximize nitrogen oxide (NO_*x*_) generation, the effect of applied frequencies ranging from 5 to 70 kHz was explored, while the applied voltage was constant (Figure 3). The result shows that ozone is the predominant product at lower frequencies. However, as the frequency reaches the optimal point of 40 kHz, the NO_*x*_ generation becomes more favorable. Beyond this threshold, while the peak positions remain unchanged, their intensities start to decrease. This reduction may be attributed to the breakdown of NO_*x*_ species into simpler compounds, particularly as higher frequencies increase the power, potentially leading to electrode overheating. Such overheating risks initiating arcing phenomena, which could reduce the efficiency of the process. Consequently, to maintain system stability and performance, it was necessary to find an optimal frequency. Therefore, we fixed the frequency at 40 kHz for all subsequent experiments to effectively control plasma chemistry and enhance NO*_x_* generation.

**Figure 4:**
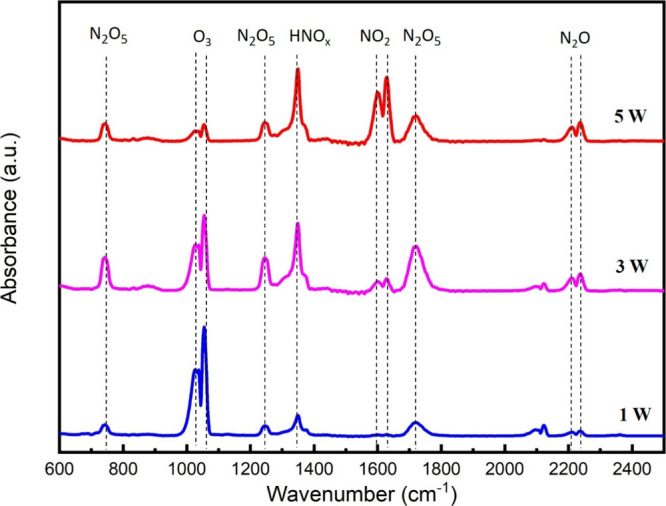
Gas-phase FTIR spectra of CDBD in the gas mixture consisting of 70 vol % Ar, 20 vol % N_2_, and 10 vol % O_2_:N_2_ (total flow rate: 80 L/h) (the frequency was adjusted at 40 kHz).

Following optimization of the applied frequency at 40 kHz, the influence of discharge power on plasma chemistry was subsequently examined. As illustrated in Figure 4, when operating at a lower discharge power, the CDBD system predominantly generated ozone (O_3_). This is supported by prominent peaks corresponding to the concentration of O_3_ at 1050 and 2100 cm^–1^ in the “Ozone Mode”. This finding emphasizes the overwhelming presence of ozone with a negligible number of NO_*x*_ species. However, as the discharge power reaches a sufficient level, the generation of nitrogen species becomes more pronounced. This change is evident in the FTIR spectra, which reveal a noticeable decrease in ozone concentration accompanied by an increase in various nitrogen oxides. Specifically, there are strong peaks for NO_2_ at 1630 cm^–1^, N_2_O at 600, 1300, and 2230 cm^–1^, N_2_O_5_ at 739 and 1717 cm^–1^, and HNO_*x*_ at 1330 and 1717 cm^–1^. An intermediate mode was observed between these two extremes (at 3 W), where the FTIR spectra captured a balanced mixture of the O_3_ and NO_*x*_ species. This mode represents a midpoint between the two extremes, featuring the spectral signatures of both ozone and nitrogen oxides in the CDBD output.

### Necessity of High Discharge Power for NO_*x*_ Species Generation

At lower power levels, the plasma lacks the requisite energy for generating atomic nitrogen and N_2_(A^3^Σ_u_^+^) metastable states due to the high dissociation energy of N_2_. Consequently, atomic oxygen generation, which requires less energy, becomes more feasible, resulting in a higher prevalence of ozone formation under these conditions. However, as the voltage is progressively increased, sufficient energy to disrupt the nitrogen bonds is provided, facilitating the production of a diverse set of reactive nitrogen species. This increase in energy triggers a competitive dynamic between reaction R1, which consumes atomic oxygen to produce ozone, and reactions R2 and R3, which utilize atomic oxygen for NO (nitric oxide) formation, subsequently leading to the formation of NO_2_ via reactions R4 and R5. Under these circumstances, the oxygen vol % in the working gas plays an essential role. With a higher oxygen concentration, the likelihood of collisions between electrons and O_2_ markedly rises, whereas the availability of nitrogen atoms and N_2_ metastable molecules diminishes, particularly enhancing the production of O_3_ through reaction R1. Therefore, to achieve a NO_*x*_-rich plasma using the working gas with high O_2_/N_2_ levels, higher discharge powers are necessary.
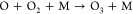
R1
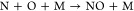
R2
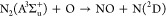
R3
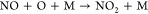
R4
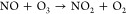
R5

### Behavior of Various Nitrogen Species across Different Power Levels

**Figure 5:**
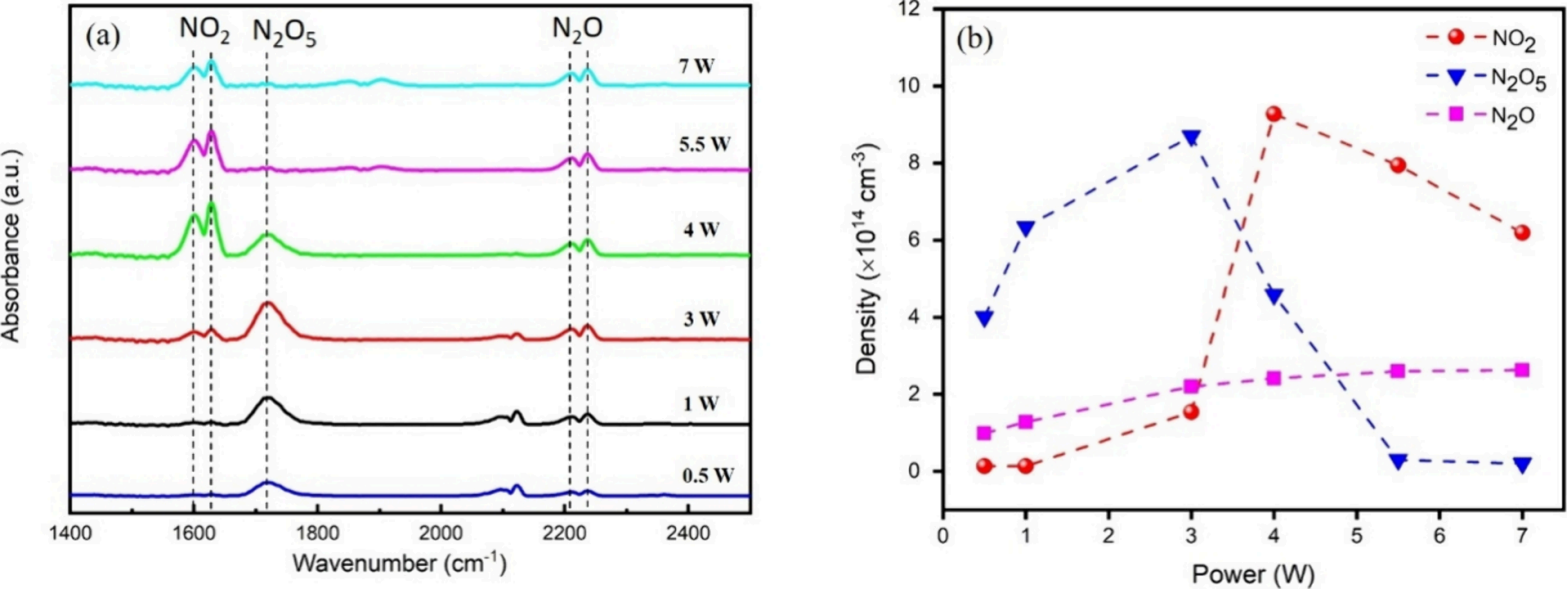
(a) Gas-phase FTIR spectra of CDBD at various discharge powers in the gas mixture consisting of 70 vol % Ar, 20 vol % N_2_, and 10 vol % O_2_ (total flow rate: 80 L/h). (b) Association of NO_2_, N_2_O_5_, and N_2_O concentrations with discharge power in the DBD system.

The formation of nitrogen dioxide (NO_2_) and the formation of dinitrogen pentoxide (N_2_O_5_) are intricately linked through a series of interdependent chemical reactions (reactions R6–R8). The formation of N_2_O_5_ critically depends on the simultaneous presence of ozone (O_3_) and nitrogen dioxide (NO_2_), as it proceeds via a reaction pathway that transforms NO_2_ and O_3_ into NO_3_, leading to the eventual formation of N_2_O_5_ (reaction R8). However, achieving high concentrations of both O_3_ and NO_2_ in plasma is challenging due to the competing discharge modes that favor either O_3_ or NO_*x*_ production. Therefore, as illustrated in Figure 5, an initial increase in power (increasing from 1 to 3 W) and the shift from the O_3_ mode to the mix mode lead to the maximal production of N_2_O_5_. However, as the power increases, the plasma energy becomes higher, providing enough energy to disrupt the N_2_O_5_ molecules back into NO_2_ and potentially into O_2_ (reaction R9). This process is favored at higher energies because it aligns with the general principle that higher plasma energies promote the dissociation of more complex molecules into simpler ones. This dissociation reaction explains why the N_2_O_5_ peak decreases and the NO_2_ peak increases with higher power (between 4 and 7 W). Nonetheless, exceeding a certain power threshold may lead to the decomposition of NO_2_ into NO (reaction R10), subsequently diminishing its concentration.

For N_2_O, lower power is also favorable, because the reaction pathway for N_2_O formation (reaction R11) typically involves the excited oxygen species (such as O(^1^D)) reacting with the first excited state of molecular nitrogen, N_2_(A^3^Σ_u_^+^), which requires less energy compared to the direct dissociation of nitrogen. At the higher discharge power and increasing N_2_(A^3^Σ_u_^+^) and O(^1^D), the probability of N_2_O conversion reactions (reactions R12–R14) rose, explaining the observed slower increase in N_2_O concentration at higher discharge power.^30^
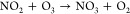
R6
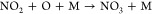
R7
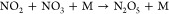
R8
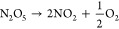
R9
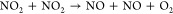
R10
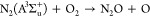
R11
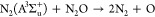
R12
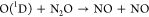
R13
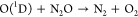
R14

### Impact of Oxygen Content in the Working Gas on NO*_x_* Concentration

**Figure 6:**
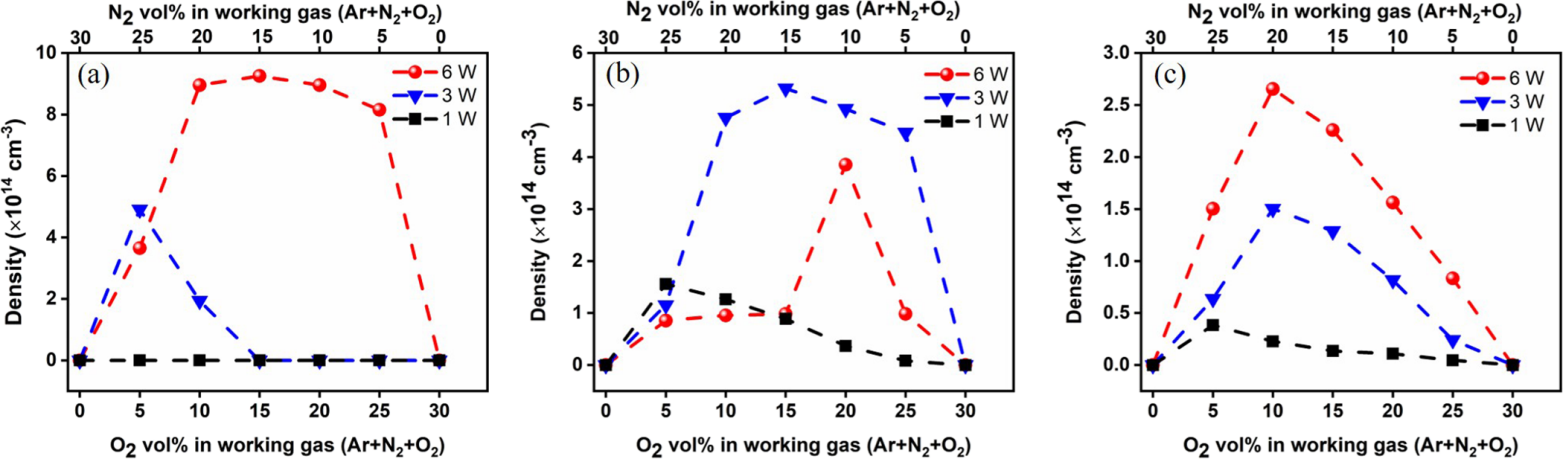
Relationship between the concentrations of (a) NO_2_, (b) N_2_O_5_, and (c) N_2_O with oxygen vol % at various discharge powers.

Figure 6 demonstrates a general parabolic pattern linking the concentrations of NO_2_, N_2_O_5_, and N_2_O with oxygen vol %. This trend indicates that the concentrations of these three species initially increase with an increase in oxygen vol %, achieve a maximum at an optimal oxygen vol %, and subsequently decline. This observed pattern could be rationalized as follows: Initially, an increase in O_2_ vol % and, subsequently, atomic oxygen contributes positively to the generation of these nitrogen species via reactions R2–R5. However, when the O_2_ vol % exceeds an optimum level because of its high electron affinity, it promotes the facile capture of electrons, consequently reducing the availability of energetic electrons, nitrogen atoms, and N_2_ metastable molecules crucial for NO and NO_2_ production. Consequently, elevated O_2_ environments impede the formation of NO and NO_2_.

However, achieving the optimal oxygen concentration to maximize the levels of NO_2_, N_2_O_5_, and N_2_O is significantly influenced by discharge power. Consequently, the O_2_ vol % should be optimized in conjunction with adjustments to the electrical properties of the DBD. Specifically, for NO_2_, when the discharge power is high enough (between 4 and 7 W), its concentration initially rises sharply, then finally becomes rather constant with an increase in O_2_ vol %, and abruptly drops to zero when the O_2_ concentration attains 30 vol % (N_2_: 0 vol %). At lower discharge powers (between 0.5 and 4 W), the NO_2_ concentration is markedly low. Therefore, the highest NO_2_ concentration is typically achieved with a discharge power of 6 W when the oxygen concentration is maintained at 10 to 20 vol %. To achieve the maximum level of N_2_O_5_, a power setting of 3 W combined with 15 vol % O_2_ is required. Concerning N_2_O, it is easier to obtain at a power range of 3–7 W when using 10 vol % of the aqueous O_2_, because with an increase in O_2_ vol %, N_2_O undergoes conversion through the following reactions:
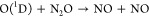
R15
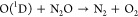
R16

### Aerosol-Assisted Plasma Polymerization

The polymerization of acrylamide into polyacrylamide served as a model reaction to evaluate the reactivity of the plasma-generated species in an aerosol state. This study also explored the potential applications and optimization of this method for removing organic pollutants or pathogens from indoor air and exhaust gases.

**Figure 7:**
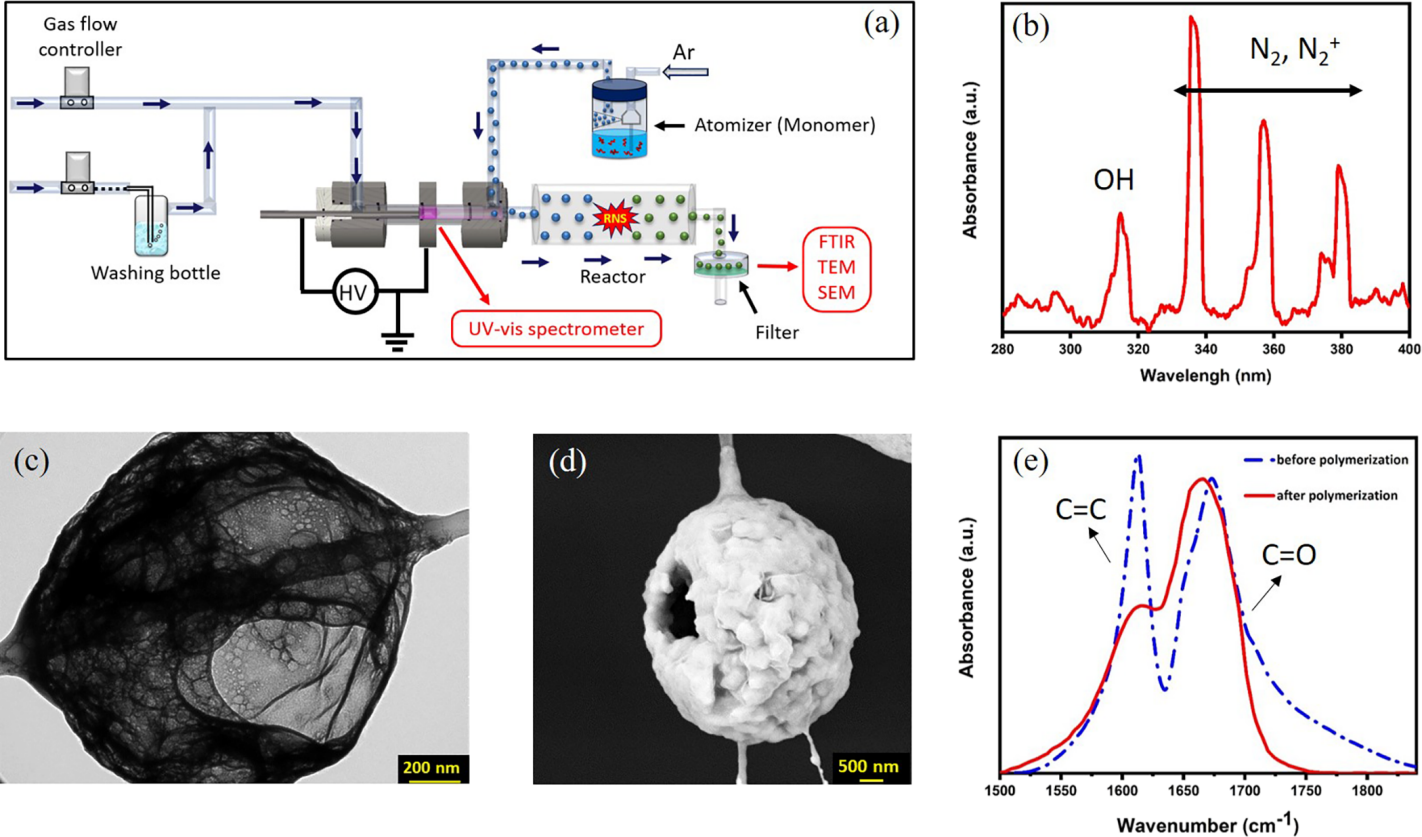
(a) Schematic of the experimental setup utilized for aerosol polymerization, (b) gas-phase UV spectra of CDBD, (c) TEM and (d) SEM image of generated PAM using aerosol polymerization, and (e) FTIR spectra of AM before and after aerosol polymerization.

The experimental setup, as shown in Figure 7a, utilized an atomizer (Topas, model ATM 220) to transform the acrylamide solution into an aerosol phase. These droplets then interacted with plasma-generated reactive species and were subsequently passed through the reactor to participate in the aerosol-polymerization process, which converted the monomer into a polymer. Short and narrow tubing was utilized to achieve a minimum residence time of 10 ms for transporting the active species from the CDBD reactor to the mixing point with the tracer aerosol.

To address the limited radiation area of the dielectric barrier discharge, a reactor was placed immediately downstream of the discharge zone. This reactor is enriched with reactive nitrogen species, including NO_2_, N_2_O, and N_2_O_5_. The primary concept is to utilize these reactive species to treat the monomer or airborne pathogens within the reactor. The average residence time in the reactor was set to 30 s to optimize the interaction between the monomer and nitrogen oxide species.

Different gas compositions were employed in this experiment, starting with research-grade humidified argon (99.999%) at a flow rate of 80 L/h. The ultraviolet–visible (UV–vis) analyses indicated that OH radicals predominated (Figure 7b). The generated polyacrylamide aerosols were collected on a specialized filter for morphological analysis using TEM and SEM (Figure 7c,d). Moreover, online FTIR was used to study the plasma-initiated polymerization yield. As illustrated in Figure 7e, there are two critical peaks for the acrylamide monomer: a C=O stretching vibration at 1733 cm^–1^ and a C=C stretching band at 1640 cm^–1^. After polymerization, a decrease in the C=C peak intensity indicated the effective transformation of monomers into polymer chains, signifying the polymerization process.^34^ The polymerization yield was quantitatively determined by comparing the intensity changes of the C=C peak relative to the C=O peak, which serves as the internal standard due to its stability during polymerization. This approach offers a straightforward method to assess the effectiveness of the polymerization process by using intrinsic molecular vibrations.
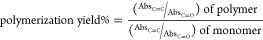


Table 1 presents the polymerization yields obtained with various gas compositions. The dominance of OH radicals results in significantly lowest yields, likely due to their short lifetime, which is estimated to be approximately 0.2 ms.^35^ However, maximum polymerization yields were achieved with a gas composition of 70 vol % Ar, 20 vol % N_2_, and 10 vol % O_2_, at a discharge power of 4 W, which could be attributed to the maximal generation of NO_2_ species under these conditions, as demonstrated by experimental results in the prior section. These findings are in good agreement with previous research^34^ indicating that the initial polymerization rate increases with increasing concentrations of NO_2_ as the initiator.

**Table 1:** Polymerization Yields Obtained with Various Gas Compositions and Discharge Powers

gas composition	discharge power (W)	predominant species in the plasma	polymerization yield (%)
humidified Ar: 100%	0.5	OH	5 ± 3
	0.7	OH	6 ± 4
	1.1	OH	6 ± 4
Ar: 70 vol %, N_2_: 25 vol %, O_2_: 5 vol %	2	O_3_, N_2_O, N_2_O_5_	8 ± 2
	4	NO_2_, N_2_O, N_2_O_5_	12 ± 4
	6	NO_2_	45 ± 5
Ar: 70 vol %, N_2_: 20 vol %, O_2_: 10 vol %	2	O_3_, N_2_O, N_2_O_5_	8 ± 5
	4	NO_2_, N_2_O, N_2_O_5_	25 ± 5
	6	NO_2_, N_2_O, N_2_O_5_	38 ± 6
Ar: 70 vol %, N_2_: 15 vol %, O_2_: 15 vol %	2	O_3_, N_2_O, N_2_O_5_	5 ± 2
	4	N_2_O, N_2_O_5_	24 ± 3
	6	NO_2_, N_2_O, N_2_O_5_	40 ± 5
Ar: 70 vol %, N_2_: 0 vol %, O_2_: 30 vol %	2	O_3_	6 ± 3
	4	O_3_	7 ± 3
	6	O_3_	6 ± 3

The brief lifespan of OH radicals presents significant challenges for experimental investigations, making direct observation and study difficult. As a result, an alternative method is required to thoroughly understand their characteristics thoroughly. In the subsequent section of this research, we shift our focus to exploring the interactions and behaviors of plasma-generated OH radicals and hydrogen peroxide (H_2_O_2_) molecules using molecular dynamics (MD) simulations. This approach allows for a detailed examination of these reactive species in a controlled computational environment.

## MD Simulations

### Setup of Molecular Dynamics Simulations

The interaction mechanisms between reactive plasma species and liquid interfaces were investigated by using molecular dynamics (MD) simulations. Employing LAMMPS,^36^ an open-source software capable of parallel processing, these simulations operationalize Newton’s equations of motion, with numerical integrators solving the dynamics over time. The ReaxFF^37^ force fields, known for their ability to model complex chemical reactivity, were pivotal in simulating the nuanced behavior of reactive oxygen and nitrogen species within liquid matrices. This computational approach is essential for understanding the detailed atomic interactions that govern plasma–liquid interfacial chemistry. A comprehensive reactive force field potential can be formulated by incorporating several distinct interaction terms, expressed as
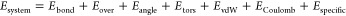
Here, the bonding interaction represents the covalent components, the van der Waals (vdW) interactions are attributed to nonbonding terms, and the Coulomb interactions represent ionic contributions. *E*_angle_ represents the deviation of the bond angle from equilibrium described by an anharmonic term, *E*_tors_ describes the four-body torsional angle strain, *E*_bond_ is a continuous function of interatomic distance and describes the energy involved in bond formation between atoms, and *E*_over_ describes an energy penalty term that prevents atoms from overcoordination, while *E*_specific_ represents specific energy contributions of the system, implying properties specific to the target system, such as lone-pair, conjugation interactions, or hydrogen binding. The ability of ReaxFF to handle a wide variety of chemical elements and its flexibility in simulating both the formation and dissociation of bonds allow for a comprehensive examination of the complex reactions that occur when plasma species interact with aqueous environments. This includes the generation, diffusion, and eventual reaction of species like OH radicals, providing valuable insights into their behavior in water.^38^

In this study, to explore the interactions of OH radicals and hydrogen peroxide (H_2_O_2_) with water molecules, we utilized a modified version of the force field developed by Monti et al. and Verlackt et al.^39,40^ This adaptation of the reactive force field is capable of simulating bond formation and dissociation, thereby providing valuable insights into the molecular reactivity. The force field has been expanded from earlier glycine parameters^41^ to include over 500 molecular systems, encompassing all amino acids and some peptides, analyzed through quantum mechanical calculations.^39^ This approach allows for effective charge distribution modeling using the electronegativity equalization method.^42^ We conducted our simulations using the ReaxFF implementation of LAMMPS software.

For the present study, water was chosen as the representative model for the liquid component of aerosol particles due to its prevalence as the primary constituent in such systems. According to the literature, respiratory droplets consist of more than 95% water at the time they are first generated.^43^ Utilizing the ReaxFF force field, we aimed to align our findings with current research while also drawing comparisons with established results from previous studies.

The methodology for preparing the water system commenced with the formation of a water column, whose length was varied up to 100 Å to attain a density consistent with that of liquid water at 1 g/cm^3^. This model system was then subjected to an equilibration period of 1000 ps at room temperature (300 K) in the canonical ensemble (*NVT*), where both temperature and volume were maintained constant using a Nosé–Hoover thermostat with a coupling constant of 25.0 fs. Following equilibration, the water column was manipulated to expand along the *z*-axis, facilitating the formation of a liquid–gas interface. This setup was crucial for simulating the interaction dynamics between the plasma species and the surface of the water, as it mirrors the conditions when plasma constituents make contact with the water in an aerosol state. To emulate the environment accurately, periodic boundary conditions were applied in the lateral directions with fixed boundaries along the *z*-axis, ensuring that the behavior of the plasma species at the interface could be studied under realistic conditions.

**Figure 8:**
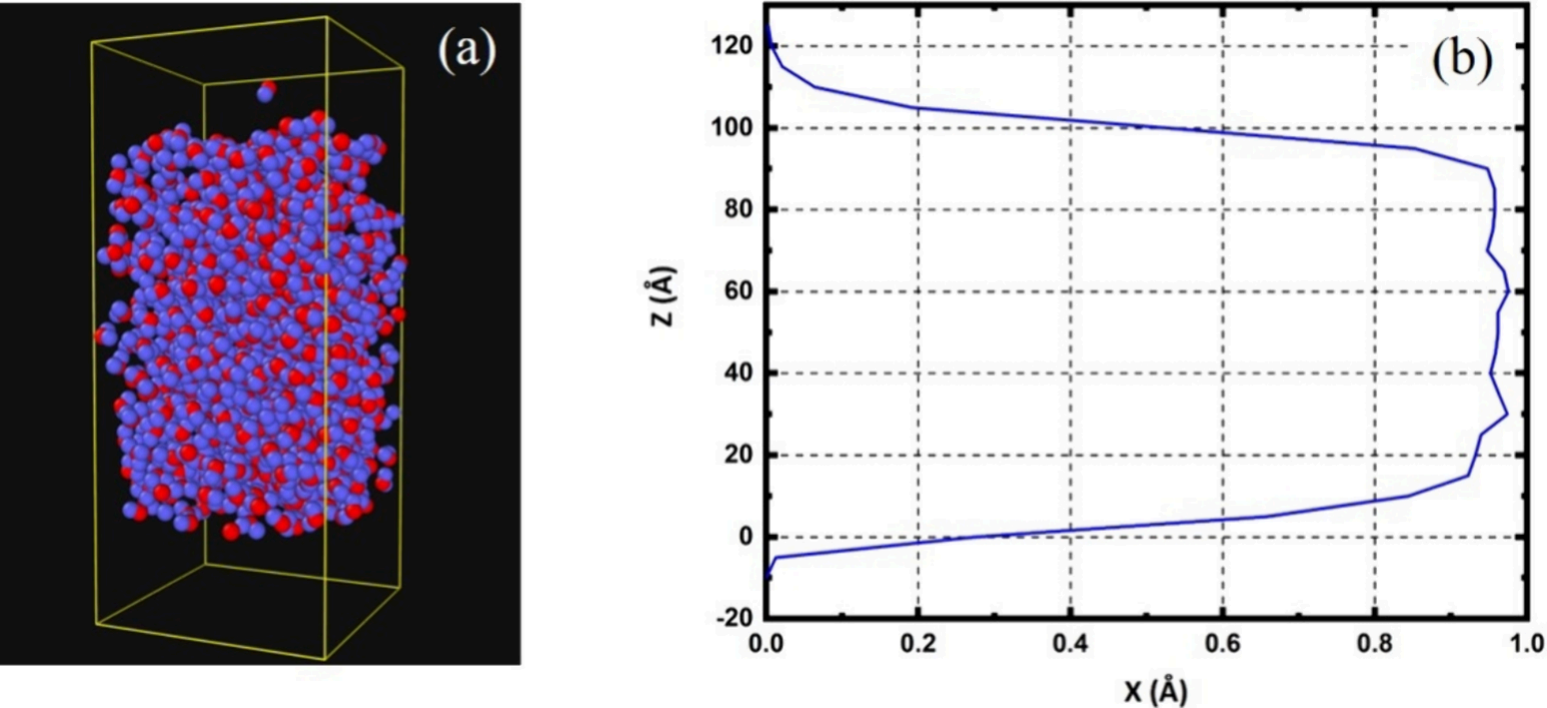
(a) Simulated water column with clear top and bottom boundaries; (b) density profile of the column showing uniform bulk water and defined interfaces.

Figure 8a depicts the simulation box, clearly delineating the water column along with its upper and lower boundaries. This model is vital for studying how plasma particles interact with water. Figure 8b presents a graph of how water density varies with the height. Sharp edges in the graph highlight these boundaries and confirm that the main water body, measuring about 100 Å in height, has a density close to that of water in its natural state. This detailed view aids in analyzing the behavior of plasma particles as they move from the gas phase and interact with the water layer.

## Simulation Results and Discussion

The investigation of the reaction mechanisms between plasma-generated species and water began by positioning these species at carefully chosen 1 nm above the water surface, each with velocities corresponding to a thermal state at 300 K. This strategy was instrumental in mitigating any preliminary interactions attributable to long-range forces, including Coulombic and van der Waals forces. The individual velocities were sampled from a Maxwellian distribution reflecting ambient conditions, and their directional orientations were uniformly randomized.

**Figure 9:**
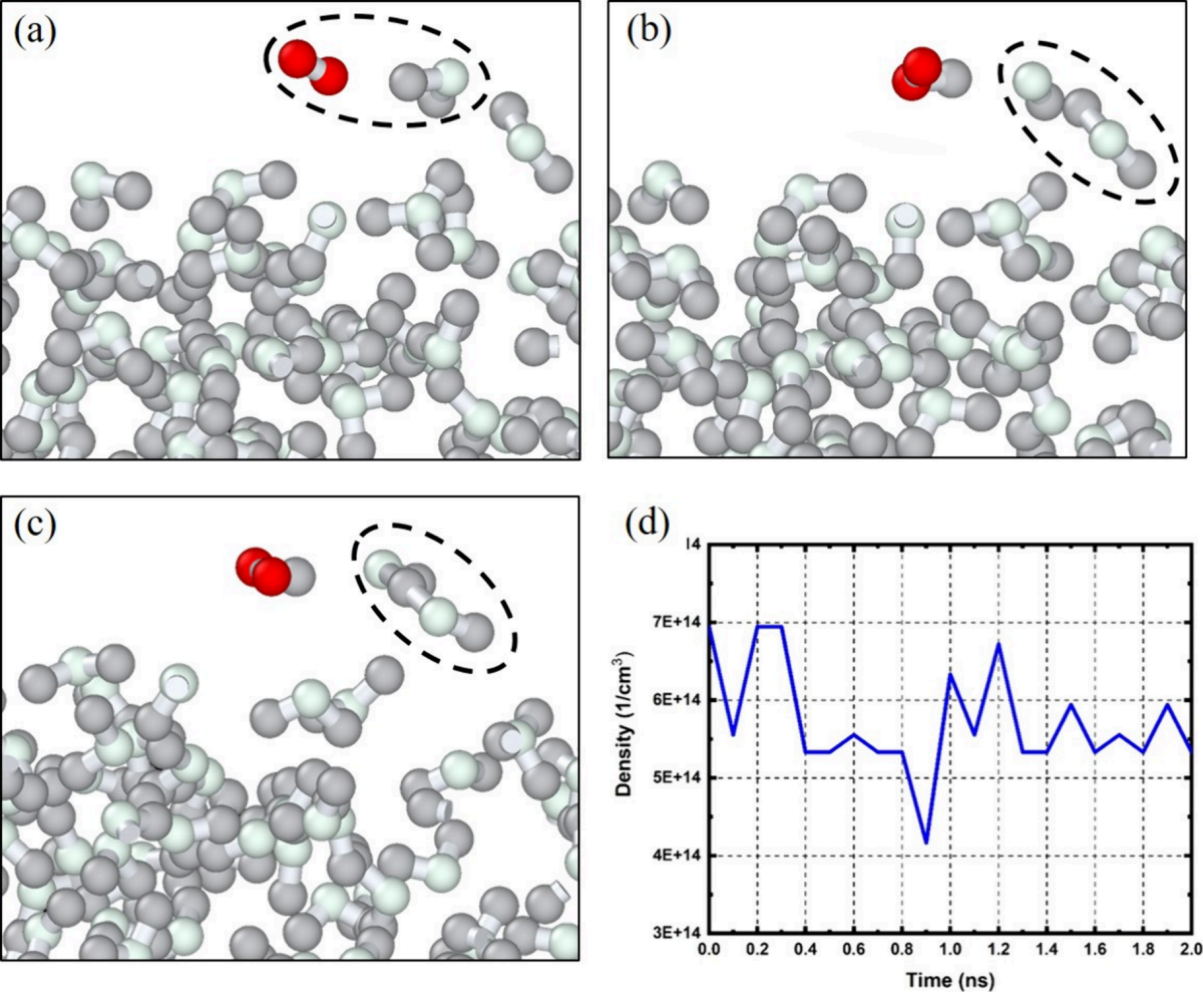
(a, b) Snapshots from MD simulations showing the interaction of OH with water, resulting in the formation of new OH radicals. (c) The reaction intermediates are shown within black dashed circles. (d) OH radical density over 2 ns, demonstrating consistent regeneration upon reacting with water. The water molecules are illustrated in grayish color and OH in red color.

In this MD investigation, the OH radical was the primary focus due to its prevalent role in plasma–water interactions. Empirical data have indicated that the OH radicals produced in cold plasma possess a minimal lifespan, typically around 0.2 ms,^35^ which may limit their suitability for direct biological applications. However, when these radicals encounter water molecules or vapor, they have the potential to engage in reactions that yield additional OH radicals. The reactive dynamics between OH radicals and water molecules are depicted in Figure 9a–c. Here, an OH radical approaches and interacts with a water molecule, creating an intermediate complex before regenerating an OH radical and a new water molecule. This cyclic reaction process, which effectively recycles the OH radicals by exchanging a hydrogen atom, is articulated in the reaction mechanism outlined in reaction R17:
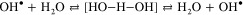
R17

Figure 9d presents the temporal evolution of the OH radical density during the molecular dynamics simulation over a 2 ns period. The relatively stable density values suggest a dynamic equilibrium where OH radicals frequently react with water molecules to form intermediate species. However, these intermediates rapidly dissociate, regenerating new OH radicals. This continuous cycle of reaction and regeneration maintains the overall population of OH radicals, indicating a persistent and self-sustaining reactive process within the simulated water environment.

**Figure 10:**
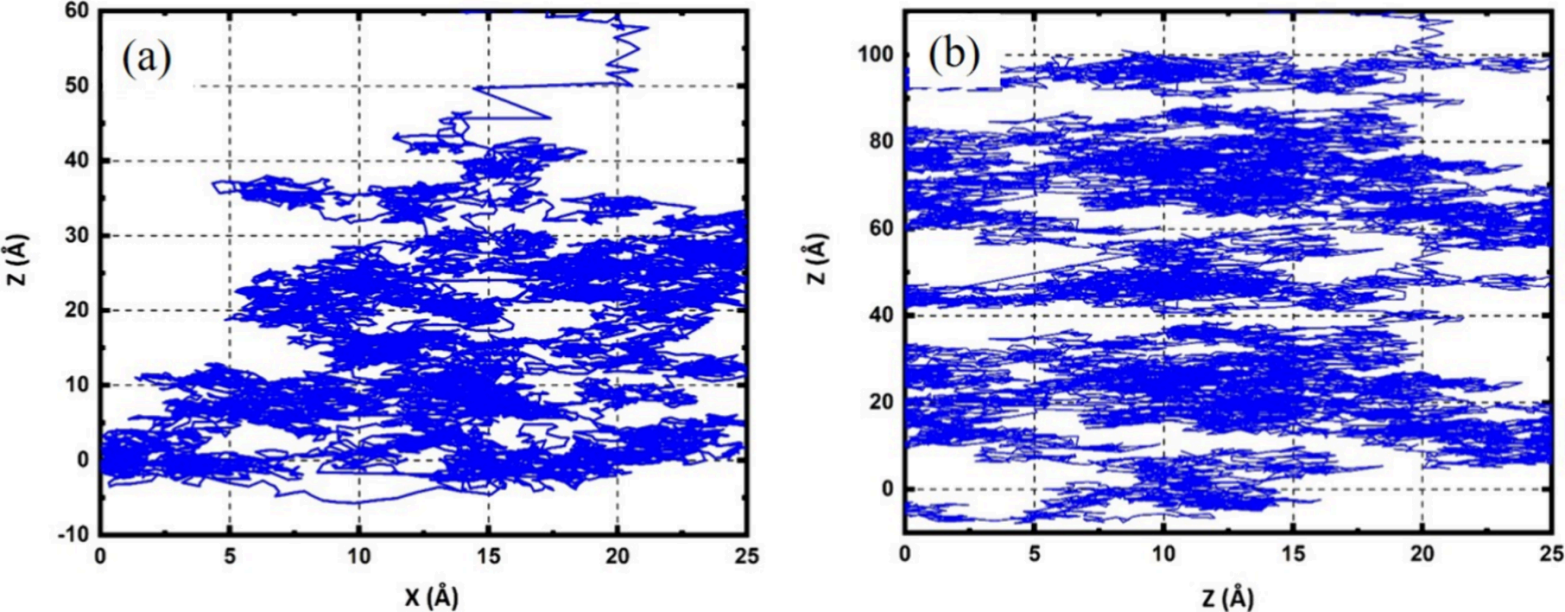
Trajectories of incident OH species within water slabs of (a) 50 and (b) 100 Å in thickness, illustrating their penetration depth over the course of the simulation. The OH radical is impacting from the top of the water slab.

Our simulations reveal the potential for OH radicals to penetrate deeply into a liquid layer, with a simulated thickness of up to 100 Å, suggesting their capacity to reach and interact with biological organisms. Figure 10 captures this phenomenon, showing the trajectory of the OH radicals within a water slab. The trajectory, marked along the *Z*-axis versus the *X*-axis, unfolds in angstroms, revealing the random and extensive movement of OH radicals. This path underscores the nonlinear and active behavior of the OH radical, interacting sporadically with the aqueous environment. The extensive movement of radicals throughout the water column not only highlights its significant mobility but also suggests a high probability of multiple interactions and potential chemical reactions with the water molecules. This behavior emphasizes the volatile nature of radicals within the simulated system.

Further analysis within our molecular dynamics simulations explored OH radical movement through water columns of 50 and 100 Å in thickness. Initially, over 2 ns, the radicals were observed to move through half of the water column, approximately 50 Å. However, with the simulation time extended to 4.1 ns, the radicals successfully reached the opposite end of the simulation box (Figure 10b). This progression illustrates that the time factor is crucial in determining the distance traveled by OH radicals. It also provides a deeper understanding of their diffusion behavior, suggesting their potential to reach and react at sites distant from their point of origin within aqueous domains.

In this study, we analyzed the behavior of OH radicals both above and within the confines of a water layer. These radicals were carefully arranged just above the water surface at various random points and ensured that they were spaced a minimum of 10 Å apart. This setup allowed for the simulation of up to seven OH radicals. We conducted the simulation over a span of 500 ps, believing this to be an adequate period to capture any potential reactions.

Our results indicated that when OH radicals are positioned approximately 20 Å above the water layer, they are prone to engage in reactions with each other, often resulting in the formation of hydrogen peroxide (H_2_O_2_) (reaction R18). The results from our simulation closely match those observed in experimental reports, particularly in the context of hydrogen peroxide production.^44^ It is believed that the primary mechanism for the generation of H_2_O_2_ is through the dimerization process of OH radicals in the reaction.^17^
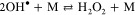
R18
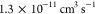


In the subsequent phase of our study, which focused on examining the behavior of OH species within the water layer, we took measures to minimize the early reactions occurring in the gas phase. This was achieved by positioning the species closer to the water surface. As a result, in the presence of water, interactions with water molecules appeared to inhibit the usual radical–radical reactions seen in the gas phase. This led to the formation of additional hydroxyl groups on the water molecules rather than hydrogen peroxide. Once solvated in the aqueous medium, the OH radicals exhibited a reduced tendency to react with each other, likely due to the stabilizing effects of solvation and the dilution effect of the water.

Additionally, the findings from our simulations indicated that the OH radical is capable of reacting with H_2_O_2_, leading to the formation of hydroperoxyl (HO_2_) radicals (reaction R19).
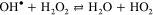
R19

Alternatively, H_2_O_2_ can also be formed in a humidified gas phase during dielectric barrier discharge (DBD) processes by using the following reactions (reactions R20–R22).
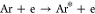
R20
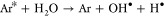
R21
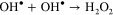
R22

**Figure 11:**
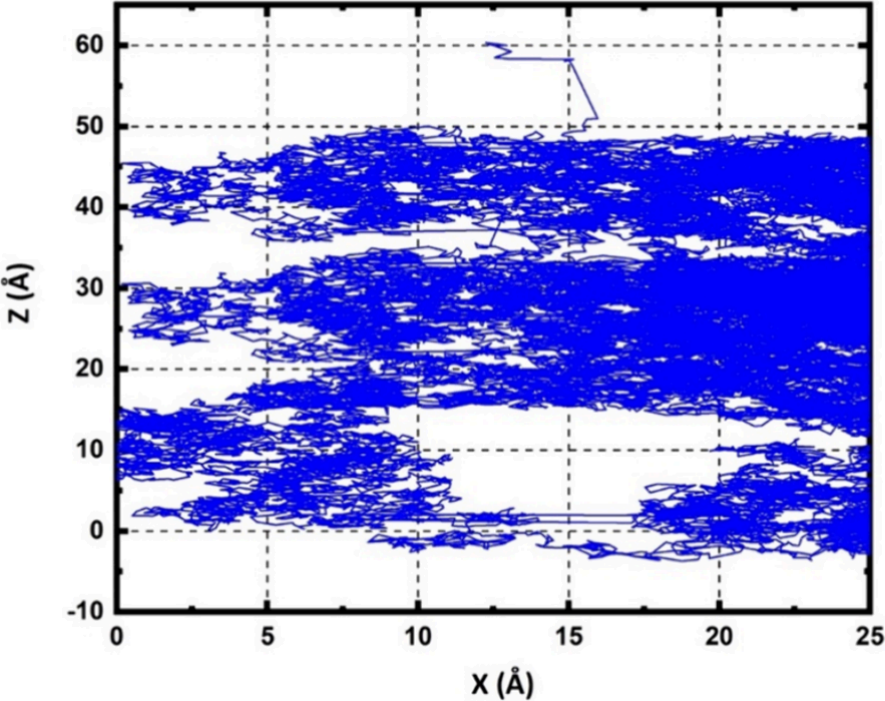
Trajectories of incident H_2_O_2_ within water slabs of 50 Å in thickness, illustrating their penetration depth over the course of the simulation. H_2_O_2_ is impacting from the top of the water slab.

In our simulations, represented in Figure 11, we discovered that hydrogen peroxide (H_2_O_2_) molecules are capable of penetrating deep into liquid layers, suggesting the potential to reach biological organisms. When immersed in water, H_2_O_2_ showed remarkable stability without any bond-breaking events, a characteristic that aligns with the findings from Moin et al., using ab initio quantum mechanical charge field molecular dynamics simulations.^45^ Their study indicated that H_2_O_2_ tends to form strong bonds with water molecules through at least four hydrogen bonds, thus enhancing its stability in aqueous environments. Furthermore, we noted that for H_2_O_2_ to permeate through a 50 Å-thick water layer, it took about 5.4 ns, significantly longer than the penetration time of OH radicals through the same layer. This difference in penetration times is attributed to the robust hydrogen bonding between H_2_O_2_ molecules and water,^45^ whereas OH radicals, capable of reacting with water to form new radicals, demonstrate a faster penetration into the water layer.

Above the water surface, particularly in the gas phase, it was observed that hydrogen peroxide (H_2_O_2_) molecules have the potential to interact with each other, leading to the formation of HO_2_ radicals. This reaction tends to occur when three H_2_O_2_ molecules come into close proximity on the water surface. In such instances, one H_2_O_2_ molecule extracts hydrogen atoms from the other two H_2_O_2_ molecules, culminating in the production of two water molecules and two HO_2_ radicals. This reaction is represented by the following equation, which is in agreement with the literature:^22,46^
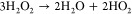
R23

### Permeation of Plasma Reactive Species through Liquid Layers

**Figure 12:**
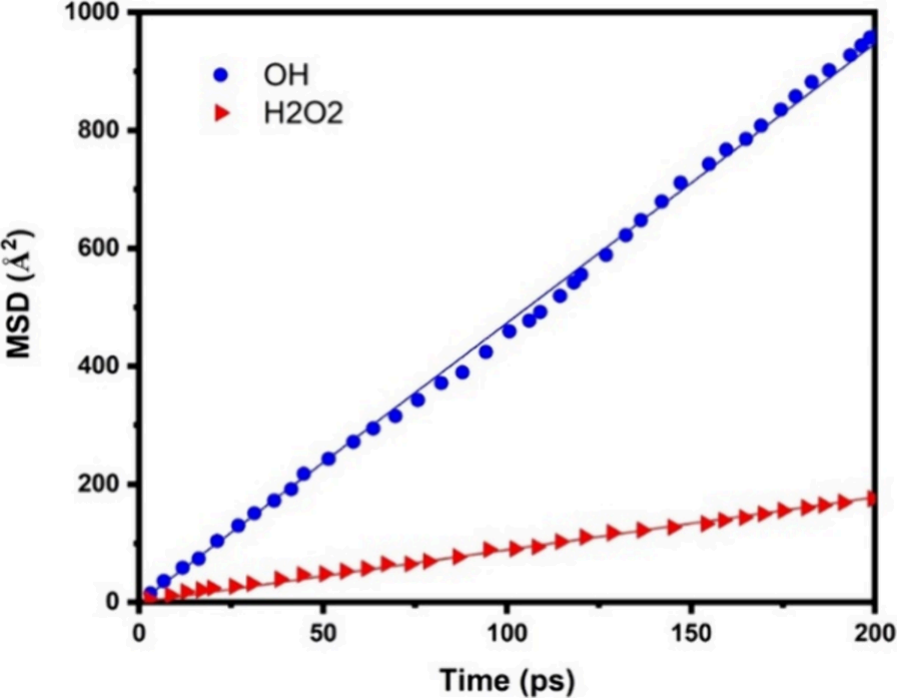
MSD of OH and H_2_O_2_ in water. The calculated diffusion coefficients are ∼0.789 and 0.148 Å^2^/ps for OH and H_2_O_2_, respectively.

The diffusion coefficients for OH and H_2_O_2_ within a water box were determined by analyzing the long-term behavior of the mean square displacement (MSD) (Figure 12). This analysis was conducted using the Einstein relation, as described in eq 1:
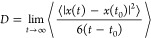
1In this context, *x*(*t*) represents the position of the molecule at time *t*. We computed the MSD within a total simulation time of 200 ps.

From this calculation, the diffusion coefficients of OH and H_2_O_2_ were determined to be 0.789 and 0.148 Å^2^/ps, respectively. These findings concur with established values reported in the literature, such as 0.71 Å^2^/ps for OH^47^ and a range of 0.13–0.15 Å^2^/ps for H_2_O_2_,^48−50^ as documented in various studies.

The experimental work focused on optimizing conditions for generating reactive nitrogen species, such as NO_2_, N_2_O_5_, and N_2_O, which have longer lifespans and are easier to analyze. The reactivity of these plasma-generated species was also evaluated through the polymerization of acrylamide into polyacrylamide, revealing that NO_2_ significantly enhanced the polymerization yields. However, our experimental observations reveal that the efficiency of the OH radical in plasma reactions may be limited by its brief lifespan. MD simulations indicate that the OH radical can penetrate the water layer and potentially stabilize within a water medium. Therefore, to take the advantage of the OH radical for environmental applications, an effective strategy is to immediately incorporate it into water droplets, enabling its stabilization. This process establishes a dynamic equilibrium that helps sustain radical activity within the water medium. These simulation results will guide our future efforts to stabilize and utilize plasma-generated OH radicals in our experimental reactions. Finally, it is important to note that while significant progress has been made, further research is needed to fully understand the interactions of plasma species, particularly NO*_x_* species, with water at the atomic level. The current force fields do not adequately describe the reactions of nitrogen species with water or biological molecules. We are actively working to develop and refine these force fields, with the goal of making them applicable to nitrogen species in the near future.
